# Rapid Transient Expression of Receptor-Binding Domain of SARS-CoV-2 and the Conserved M2e Peptide of Influenza A Virus Linked to Flagellin in *Nicotiana benthamiana* Plants Using Self-Replicating Viral Vector

**DOI:** 10.3390/plants11243425

**Published:** 2022-12-08

**Authors:** Eugenia S. Mardanova, Roman Y. Kotlyarov, Nikolai V. Ravin

**Affiliations:** Institute of Bioengineering, Research Center of Biotechnology of the Russian Academy of Sciences, 119071 Moscow, Russia

**Keywords:** plant-produced vaccine, transient expression, SARS-CoV-2, influenza A, flagellin

## Abstract

The development of recombinant vaccines against SARS-CoV-2 and influenza A is an important task. The combination of the conserved influenza A antigen, the extracellular domain of the transmembrane protein M2 (M2e), and the receptor-binding domain of the SARS-CoV-2 spike glycoprotein (RBD) provides the opportunity to develop a bivalent vaccine against these infections. The fusion of antigens with bacterial flagellin, the ligand for Toll-like receptor 5 and potent mucosal adjuvant, may increase the immunogenicity of the candidate vaccines and enable intranasal immunization. In this study, we report the transient expression of RBD alone, RBD coupled with four copies of M2e, and fusions of RBD and RBD-4M2e with flagellin in *Nicotiana benthamiana* plants using the self-replicating potato virus X-based vector pEff. The yields of purified recombinant proteins per gram of fresh leaf tissue were about 20 µg for RBD, 50–60 µg for RBD-4M2e and the fusion of RBD with flagellin, and about 90 µg for RBD-4M2e fused to flagellin. Targeting to the endoplasmic reticulum enabled the production of glycosylated recombinant proteins comprising RBD. Our results show that plant-produced RBD and RBD-4M2e could be further used for the development of subunit vaccines against COVID-19 and a bivalent vaccine against COVID-19 and influenza A, while flagellin fusions could be used for the development of intranasal vaccines.

## 1. Introduction

Expression platforms that can respond quickly to emerging infections and are easy to use are essential for the production of preventive or therapeutic vaccines to address emerging biosecurity and global health challenges. Plant-based systems for the expression of recombinant proteins have a number of inherent advantages over bacterial, yeast, and animal cell systems in terms of speed, cost, scalability, and safety. Additionally, plants are capable of posttranslational modifications that could be crucially important for the stability, transport, and biological activity of proteins. A promising way to express target proteins in plants is transient expression. Transient expression systems are fast and simple, in contrast to the traditional laborious and time-consuming procedure for creating stable transgenic lines [1,2,3]. The transient transfection of plant tissues appeared as an alternative method to stable transgenesis at the turn of the century, opening up new possibilities for plant biotechnology in protein synthesis, metabolic engineering, and synthetic biology. Agroinfiltration, the process of introducing a plasmid vector with the help of special strains of *Agrobacterium tumefaciens* into individual leaves or whole plants, leads to the spread of T-DNA to all cells in the infiltrated area. This method has three important advantages for the expression of recombinant proteins: it allows the process to be scaled up quickly and easily; simplifies the creation of gene expression cassettes; and it separates host cell creation from plant growth and biomass accumulation [4,5,6].

The family Coronaviridae includes a large group of viruses known as coronaviruses (CoVs) that cause a wide range of diseases. Humans, birds, sheep, mice, bats, and numerous other wild species become infected with CoVs in the gastrointestinal, respiratory, hepatic, and central nervous systems [7,8]. Since its discovery in China at the end of 2019, the severe acute respiratory syndrome coronavirus 2 (SARS-CoV-2) has been related to more than 629 million confirmed cases and 6.5 million deaths worldwide up to 9 November 2022 [9]. Although various pharmacological treatments have been developed, mass immunization remains the most effective method to prevent new coronavirus outbreaks [10].

SARS-CoV-2 has a single-stranded, positive-sense RNA genome of about 29.9 kb in size. The genome encodes four structural (S, E, M, and N) and up to six accessory proteins [11]. The spike glycoprotein (S) assembles into homotrimers, which are then displayed on the virion surface and give it the coronavirus’s characteristic crown-like look. The S protein is the main antigen capable of inducing protective immune responses, and is required for the interaction of the virus with the cellular receptor during virus entry. The S protein has thus far been the most promising target protein for the creation of vaccines [12,13]. The analysis of the structure of the S protein allowed identification of the receptor-binding domain (319–541 a.a.) responsible for direct interaction with the host angiotensin-converting enzyme 2 (ACE2) receptor. Thus, the receptor-binding domain (RBD) plays a key role in the process of virus entry into the cell [14,15]. The RBD is a promising vaccine candidate, and many vaccines are in various stages of clinical trials [15]. Neutralizing antibodies that prevent the virus binding to the receptor are most commonly directed against the RBD [16]. SARS-CoV-2, like other RNA viruses, is subject to a fairly rapid process of mutagenesis due to the low fidelity of its RNA-dependent RNA polymerase. The most frequent and highly contagious strains include such families as Alpha (B.1.1.7), Beta (B.1.351), Gamma (P.1), Delta (B.1.617.2), and Omicron (B.1.1.529). At the time this work started, the Delta strain was dominant in the world and therefore we used the RBD sequence from this strain in our work [17]. The sequence of the RBD domain of the Delta strain differs in three amino acid residues compared to the sequence of the original Wuhan strain.

Several RBD-based candidate vaccines are in various stages of clinical and preclinical trials [15,16]. There is also an example of a subunit RBD-based vaccine produced in plants, for which phase 1 and 2 trials are currently ending (NCT04473690 conducted by Kentucky BioProcessing). There are also a number of publications showing not only the possibility of using RBD as part of candidate vaccine proteins, but also confirming their immunogenic properties in different models [18,19,20,21,22,23,24,25,26]. The S protein has 22 potential canonical N-glycosylation sites (N-X-S/T, where X could be any amino acid except P). The RBD region contains two experimentally verified (N331 and N343) N-glycosylation sites [27,28]. N-glycans are important for protein folding and the efficient production of RBD as a functional protein [25].

Influenza viruses trigger seasonal epidemics and pose a serious danger to public health all over the world. Due to the relatively high rate of occurrence of point mutations in surface glycoproteins (antigenic drift), new antigenic variants appear almost every year. In addition to annual epidemics, every few decades there are pandemic influenza outbreaks causing millions of deaths worldwide. Pandemic strains generally arise from the phenomenon of the reassortment of genome sections of two or more strains of different origin (antigenic shift). The resulting virus differs significantly from the parent strains and, as a consequence, the population has no meaningful immunity against it. This causes higher levels of morbidity, severity of progression, and mortality [29,30]. Mass vaccination remains the most effective way to deal with flu outbreaks. Most influenza vaccines are based on the most abundant surface antigens, hemagglutinin and neuraminidase. However, these proteins are the most frequent targets for antigenic drift and shift and, therefore, such vaccines need to be renewed almost every year to meet the antigenic specificity of newly emerging strains. Therefore, efforts are being made to produce universal influenza vaccines that offer wide protection and long-lasting immunity [29,31]. Such universal vaccines should be based on conserved viral antigens. The extracellular domain of the membrane protein M2 (M2e) is a short (23 a.a.) peptide whose sequence is highly conserved in all the human isolates of the influenza A virus and differs in few positions in the strains of animal origin [32]. The immunogenicity of native M2e is low, but it can be improved by fusing M2e to a highly immunogenic carrier or adjuvant. The first study in which M2e was presented on the surface of virus-like particles formed by the core antigen of the hepatitis B virus dates back to 1999 [33], and a number of further studies demonstrated that M2e-based vaccines could be highly immunogenic and protect against infection (reviewed in [34,35]). Examples of recent clinical trials of M2e-containing candidate vaccines include Uniflu (NCT03789539) and Tandiflu1 [36,37].

Recombinant vaccines often require an adjuvant, due to the low immunogenicity of individual peptides or proteins as opposed to complete viral particles [38]. In particular, the target antigen could be linked to a highly immunogenic carrier protein acting as an adjuvant. These kinds of adjuvants include Toll-like receptors (TLRs) [39]. By appropriately arming dendritic and other antigen-presenting cells, activating crucial costimulatory and regulatory systems, and encouraging antigen presentation, TLRs have been found to play a crucial role in directing the adaptive immune response [40]. The bacterial flagellin belongs to this class of proteins, being a ligand for TLR5. Flagellin is highly effective as a mucosal adjuvant, which enables non-invasive intranasal vaccination. Flagellin has proven to be an effective platform and adjuvant for numerous vaccines in a variety of infection models, including influenza [41,42,43].

The seasonal influenza A virus and SARS-CoV-2, which has tended to be seasonal, are important pathogens in terms of bivalent vaccine development. The M2e peptide is already an established target for the development of recombinant “universal” vaccines against influenza A, whereas the RBD of the coronavirus is a promising antigen for coronavirus candidate vaccines. The combination of influenza A and SARS-CoV-2 antigens in one vaccine would allow for the prevention of diseases quickly and conveniently. Additionally, considering the ability of flagellin to act as a mucosal adjuvant suitable for intranasal immunization and the important role of nasal mucosa in the transmission and the clinical progression of both influenza and SARS-CoV-2, the use of flagellin as a carrier could be an effective strategy for immunization against SARS-CoV-2 and influenza A. Therefore, in this study we designed fusion proteins comprising the M2e of the influenza A virus and the RDB of SARS-CoV2 alone or fused to the flagellin of *Salmonella typhimurium.* We demonstrated that these proteins could be quickly and efficiently produced in *Nicotiana benthamiana* plants using a transient expression system.

## 2. Results

### 2.1. Recombinant Proteins and Viral Vectors

Previously, we reported the efficient transient expression of the RBD peptide (319–524 a.a., strain Wuhan-Hu-1) fused to flagellin in *N. benthamiana* plants [26] using viral vector pEff [44]. Fusion Flg-RBD protein was expressed at the level of 110–140 μg/g of fresh leaf tissue, but was insoluble. In this study, we implemented several modifications of the target proteins to improve the efficiency of expression. First, the nucleotide sequence encoding the RBD (319 to 541 a.a. of the Delta strain of SARS-CoV-2) was codon-optimized for expression in *N. benthamiana*. Second, a flexible glycine-rich 19S linker [45] was inserted between the flagellin and RBD to facilitate the proper folding of the hybrid protein. Third, we modified the pEff expression vector to encode the 23 a.a. signal peptide (SP) from *Phaseolus vulgaris* at the N-terminus and the endoplasmic reticulum retention signal sequence HDEL at the C-terminus of the target protein [46]. These signal sequences were included for targeting the recombinant protein to the endoplasmic reticulum and its retention in this compartment. In addition, 8-histidine tag and GGS linker were inserted between the target protein and the HDEL sequence to enable purification by metal affinity chromatography.

**Figure 1:**
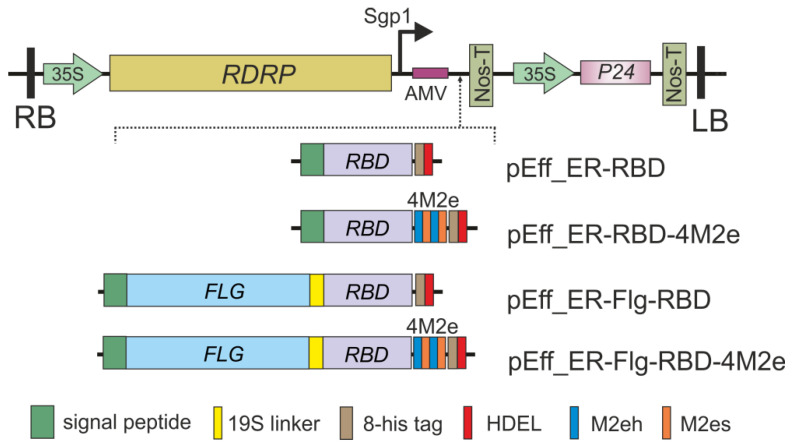
Scheme of the expression vectors. RDRP, RNA-dependent RNA polymerase gene; Sgp1, the first promoter of subgenomic RNA of PVX; AMV, translational enhancer from alfalfa mosaic virus; 35S, promoter of the cauliflower mosaic virus RNA; Nos-T, terminator of the *A. tumefaciens* nopaline synthase gene; P24, suppressor of silencing from grapevine leafroll-associated virus-2; Flg, flagellin of *S. typhimurium*; RBD, the sequence of receptor-binding domain of SARS-CoV-2; 4M2e, the sequence of four tandem copies of M2e peptide; SP, signal peptide; HDEL, ER retention signal; RB and LB, the right and left borders of T-DNA region.

Four recombinant proteins were designed for expression in *N. benthamiana* (Figure 1): the RBD peptide of SARS-CoV-2 alone, RBD peptide fused with four copies of the M2e peptide of the influenza A virus (RBD-4M2e), as well as the RBD and RBD-4M2e genetically linked to the C-terminus of *S. typhimurium* flagellin (Flg-RBD and Flg-RBD-4M2e). The 4M2e sequence comprises two copies of the human consensus M2e sequence and two copies of M2e of the pandemic influenza virus strain A/H1N1pdm09. Individual copies of the M2e peptides were separated by GGGSG linkers to facilitate the folding of the fusion protein. The pEff viral vector [44] was used for the expression of the target proteins in the *N. benthamiana* plants (Figure 1).

### 2.2. Expression and Purification of Recombinant Proteins

Recombinant vectors pEff_ER-RBD, pEff_ER-RBD-4M2e, pEff_ER-Flg-RBD, and pEff_ER-Flg-RBD-4M2e were introduced into the *A. tumefaciens* strain GV3101 by electroporation. The obtained strains were used for the infiltration of the leaves of the *N. benthamiana* plants. After the delivery of agrobacteria into the plant tissue, T-DNA with an expression cassette was transferred to the plant cells, followed by the replication of the viral vector RNA, the synthesis of the subgenomic RNA encoding the target gene, and its translation with the production of the target protein. Leaf tissues for the isolation of the protein samples were harvested four days after agroinfiltration since in the following days leaves developed necrotic phenotypes. The necrosis was also observed in the case of the expression of RBD alone [18] and the Flg-RBD fusion protein in plants [26].

**Figure 2:**
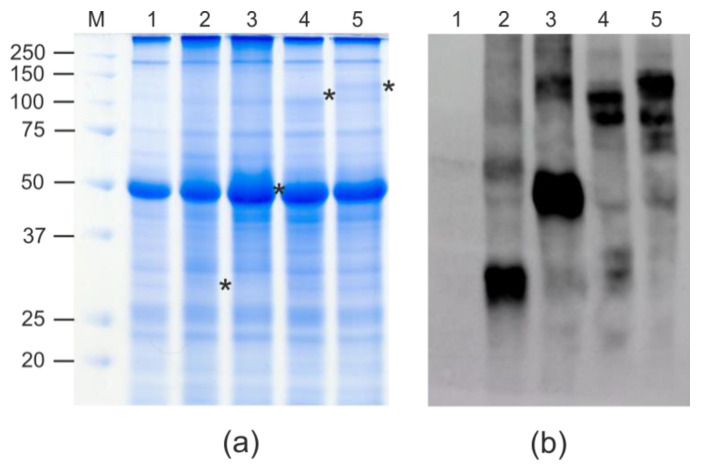
Expression of recombinant proteins in *N. benthamiana* plants. Coomassie brilliant blue-stained gel (**a**) and Western blotting with antibodies against RBD (**b**) of proteins isolated from plants. M, molecular weight marker (kD); Lanes: 1, total proteins isolated from the non-infiltrated leaf; 2, total proteins isolated from leaf infiltrated with pEff_ER-RBD; 3, total proteins isolated from leaf infiltrated with pEff_ER-RBD-4M2e; 4, total proteins isolated from leaf infiltrated with pEff_ER-Flg-RBD; 5, total proteins isolated from leaf infiltrated with pEff_ER-Flg-RBD-4M2e. Positions of RBD (calculated molecular weight 30 kD), RBD-4M2e (43 kD), Flg-RBD (83 kD), and Flg-RBD-4M2e (95 kD) proteins are shown by asterisks.

**Figure 3:**
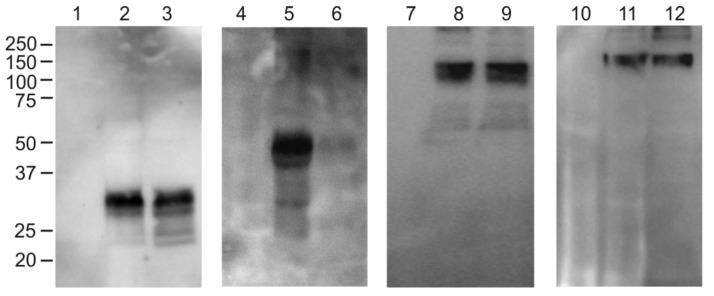
Analysis of the solubility of recombinant proteins expressed in *N. benthamiana*. Lanes: 1, 4, 7, 10, total proteins isolated from non-infiltrated leaves; 2, total proteins from leaf infiltrated with pEff_ER-RBD; 3, soluble proteins from leaf infiltrated with pEff_ER-RBD; 5, total proteins from leaf infiltrated with pEff_ER-RBD-4M2e; 6, soluble proteins from leaf infiltrated with pEff_ER-RBD-4M2e; 8, total proteins from leaf infiltrated with pEff_ER-Flg-RBD; 9, soluble proteins from leaf infiltrated with pEff_ER-Flg-RBD; 11, total proteins from leaf infiltrated with pEff_ER-Flg-RBD-4M2e; 12, soluble proteins from leaf infiltrated with pEff_ER-Flg-RBD-4M2e. Western blotting was performed with antibodies against RBD (lanes 1–6) or flagellin (lanes 7–12).

The protein samples from the agroinfiltrated leaves were analyzed using SDS-PAGE and Western blotting (Figure 2). SDS-PAGE and the Western blotting of the total protein samples showed that all four proteins were expressed and specifically revealed with the antibodies against RBD (Figure 2). An analysis of the total proteins and the soluble fraction by Western blotting showed that RBD-4M2e protein was predominantly insoluble, whereas RBD, Flg-RBD, and Flg-RBD-4M2e were present in the soluble fraction (Figure 3). The expression level of flagellin-fused proteins was about 100–150 μg/g of fresh leaf tissue, RBD alone—about 50–60 μg/g of fresh leaf tissue, and the expression level of about 150 μg/g of fresh leaf tissue was observed for the RBD-4M2e protein.

The purification of the recombinant RBD-4M2e and RBD proteins was performed using metal-affinity chromatography under denaturing and native conditions, respectively, in accordance with their solubility. Flagellin-based proteins were isolated under denaturing conditions to minimize their proteolytic degradation [42]. After elution from the sorbent, the purified protein samples were dialyzed against phosphate-buffered saline (PBS). After the dialysis, all purified RBD proteins remained soluble. The final yield of RBD after purification was about 20 μg/g of fresh leaf biomass, which is comparable with the results of Mamedov et al. [21]. The purification yield of RBD-4M2e, Flg-RBD, and Flg-RBD-4M2e was about 60, 60, and 90 μg/g of fresh leaf tissue.

**Figure 4:**
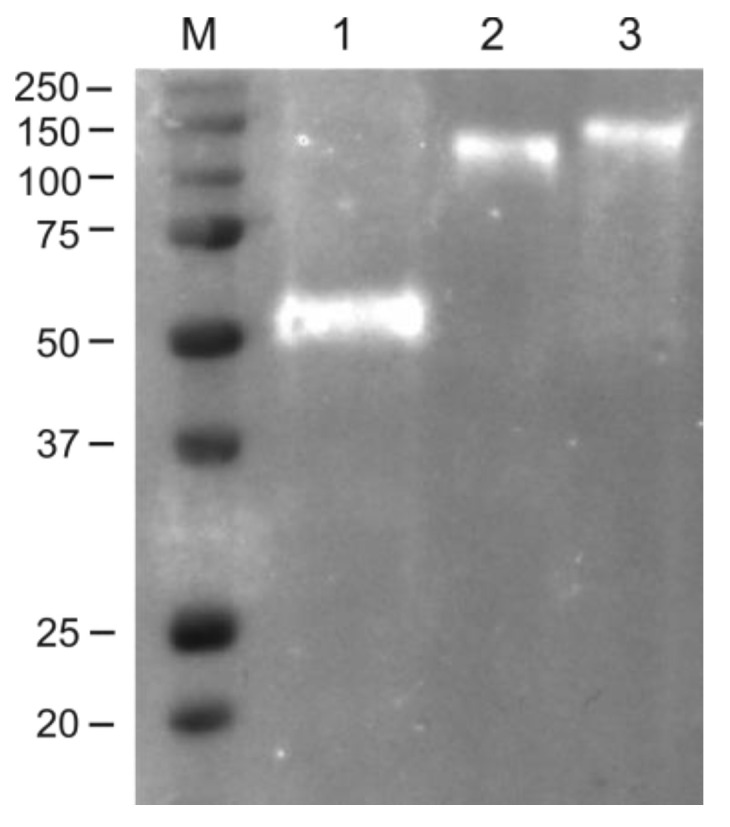
Glycosylation of plant-produced proteins. M, molecular weight marker (kD); Lane 1, purified RBD-4M2e; lane 2, purified Flg-RBD; lane 3, purified Flg-RBD-4M2e.

The RBD peptide contains two experimentally verified N-glycosylation sites, N331 and N343, which are fully glycosylated when expressed in heterologous expression systems [27,28,47]. Therefore, the recombinant RBD-4M2e, Flg-RBD, and Flg-RBD-4M2e proteins were subjected to glycan detection to demonstrate their in vivo glycosylation. As shown in Figure 4, N-glycans were detected in all three plant-produced recombinant proteins.

## 3. Discussion

Traditional influenza A and newly emerging COVID-19 are at present probably the two most important respiratory infection diseases demanding the development and fast large-scale production of cheap and effective vaccines. The purpose of our study was to develop a plant-based expression system for the production of potential vaccine candidates against both influenza A and SARS-CoV-2 at the same time.

The M2e peptide of the influenza A virus is one of the most promising candidates for the development of a recombinant influenza vaccine since its sequence is highly conserved in all human isolates and differs in only a few amino acids in strains of animal origin [33,48]. Likewise, the RBD region of the S protein of SARS-CoV-2 could be a promising target for the development of recombinant vaccines against COVID-19 [15,16]. Previous studies have shown that the immunogenicity of native M2e is poor, but it can be increased by its fusion to highly immunogenic carriers or adjuvants [33,34,49]. In this study, we developed a plant-based expression system for the production of the RBD and M2e linked to bacterial flagellin, known to be a potent mucosal adjuvant.

For the expression of recombinant proteins, we used self-replicating viral vector pEff. In the previous pilot study, we used this vector to express recombinant protein containing bacterial flagellin fused to RBD [26]. Although this protein was relatively well expressed (110–140 μg/g of fresh leaf biomass), it appeared to be fully insoluble. Therefore, in the present study we not only constructed recombinant proteins containing both M2e and RBD, but also optimized the expression system itself. These improvements included the introduction of glycine-rich linkers for the better spatial separation of the flagellin and fusion partners, which may facilitate the proper folding of the hybrid protein, and targeting of the hybrid protein to the endoplasmic reticulum. In addition, for the RBD-coding sequence we performed the codon optimization for *N. benthamiana*. These improvements enabled us to produce both RBD alone and flagellin fusions in a soluble form at a relatively high level. The RBD-4M2e protein remained insoluble; perhaps the reason lies in the presence of multiple cysteine residues (two in M2e and nine in RBD), which can cause the formation of disulfide bonds and protein aggregation, as reported previously for M2e [50]. Moreover, the targeting of recombinant proteins to the endoplasmic reticulum in plants enabled their production in glycosylated form [51], as observed in our study. N-glycosylation was shown to be critical for the production of correctly folded functional recombinant RBD variants [25].

The prospects for the development of a bivalent vaccine against SARS-CoV-2 and influenza A are also evidenced by the results of a recent study in which the SARS-CoV-2 spike protein or the RBD domain was co-expressed with four copies of M2e fused with the Ebola glycoprotein DC-targeting/activation domain in mammalian cells using a recombinant vesicular stomatitis virus vector [52]. This study showed that such bivalent vaccine candidates induced efficient humoral and cellular immune responses against both the SARS-CoV-2 spike protein and the influenza M2 protein in immunized animals [52].

Overall, this study demonstrated that the fast and relatively high-level expression of recombinant proteins comprising Flg and RBD or both RBD and M2e in plants is feasible. The latter protein could be a basis for the development of a bivalent vaccine against COVID-19 and influenza A. Considering the important role of nasal mucosa in the transmission and the clinical progression of both influenza and SARS-CoV-2, immunization using flagellin-based intranasal vaccines could be an effective strategy for immunization against SARS-CoV-2 and influenza A. Our efforts to evaluate the immunogenic properties of flagellin-based RBD and RBD/M2e fusions are presently underway.

## 4. Materials and Methods

### 4.1. Gene Cloning and Construction of Expression Vectors

The pEff vector contains the gene encoding an RNA-dependent RNA polymerase of PVX, promoter of the first viral subgenomic RNA, translation enhancer from the alfalfa mosaic virus, the *gfp* gene flanked by unique restriction site *Asc*I and *Sma*I, followed by the 3′ terminal part of the coat protein gene and the 3′-untranslated region of the PVX genome. This expression cassette is located downstream of the 35S promoter. All these genetic elements are located within the T-DNA region of a binary vector that can be maintained both in *Escherichia coli* and *Agrobacterium tumefaciens*. RNA-dependent RNA polymerase enables replication of the vector in a plant cell, which ensures a high level of expression of the target protein [44].

Nucleotide sequences encoding the ER-targeting signal peptide from *Phaseolus vulgaris* (MIMASSKLLSLALFLALLSHANS), the RBD region of the S protein (319–541 a.a.) of SARS-CoV-2 strain Delta spike protein (GenBank QZC92021.1), 8-histidine tag, and plant-specific ER retention signal HDEL, was optimized for expression in *N. benthamiana* using online Codon Optimization Tool (https://eu.idtdna.com/pages/tools/codon-optimization-tool accessed on 4 November 2022) and synthesizing in vitro (Evrogen Inc., Moscow, Russia). The resulting sequence was inserted into pEff-GFP expression vector [44] instead of GFP, resulting in pEff_ER-RBD expression vector (Figure 1).

Nucleotide sequence encoding the flagellin gene of *S. typhimurium* followed by 19S linker (GTSGSSGSGSGGSGSGGGG) was amplified by PCR using primers Flg_NruI_F (ATA TCG CGA GCA CAA GTA ATC AAC ACT AA) and Flg_19S_SacI_R (TAT GAG CTC TCC ACC ACC TCC AGA CCC AGA CCC GCC GCT ACC ACT ACC TGA GGA TCC AGA TGT ACC CAC CTG GTT AGC CTG CGC CA) using previously obtained plasmid pEff-Flg-RBD [26] as a template. The resulting PCR fragment was cloned in pEff_ER-RBD vector at *Nru*I/*Sac*I restriction sites resulting in expression vector pEff_ER-Flg-RBD.

Four copies of M2e comprising two copies of the human consensus M2e sequence (M2eh, SLLTEVETPIRNEWGCRCNDSSD) and two copies of M2e of the pandemic influenza virus strain A/H1N1pdm09 (M2es, SLLTEVETPTRSEWECRCSDSSD) arranged as M2eh-M2es-M2eh-M2es were fused to the C-terminus of RBD. The 4M2e-coding sequence was excised as a *Stu*I/*Sma*I fragment from plasmid pQE30_Flg4M2e(2h2s) [43] and cloned at the *Sma*I site of pEff_ER-RBD and pEff_ER-Flg-RBD resulting in expression vectors pEff_ER-RBD-4M2e and pEff_ER-Flg-RBD-4M2e, respectively.

These expression vectors were then transferred into *A. tumefaciens* strain GV3101 by electroporation. To express recombinant proteins in *N. benthamiana* plants, agrobacteria harboring pEff-based vectors were infiltrated into *N. benthamiana* leaves as described previously [26]. Plants were harvested at 4 days post infiltration.

### 4.2. SDS-PAGE and Western Blotting

Pieces of agroinfiltrated leaves (~10 mg) were homogenized in 50 μL of the extraction buffer (50 mM NaH_2_PO_4_, 300 mM NaCI, pH 8.0). To obtain soluble protein fraction, the mixture was subjected to centrifugation at 14,000× *g* for 10 min and the supernatant was collected. An equal volume of loading buffer for SDS-PAGE (20% glycerol, 5% SDS, 62.5 mM Tris pH 6.8, 0.5% bromphenol blue, 5% β-mercaptoethanol) was added either to the initial suspension (total protein fraction) or to the supernatant (soluble protein fraction). Then, 10 µL of the obtained solution (corresponding to about 1 mg of leaf biomass) was analyzed by SDS-PAGE (10%). After electrophoresis, the bands were visualized by staining of the gel with One-Step Blue Protein Gel Stain (BIOTIUM, Fremont, CA, USA).

For Western blotting, the proteins separated in the SDS-PAGE gel were transferred onto a Hybond-P membrane (GE Healthcare, New York, NY, USA) employing the Trans-Blot Turbo Transfer System (Bio-Rad Laboratories, Hercules, CA, USA). Then, the membrane was blocked with a 5% (*w*/*v*) solution of dry milk in TBS-T (20 mM Tris pH 8.0, 150 mM NaCI, 0.1% Tween 20) buffer for 1 h at room temperature and subsequently incubated with mouse polyclonal anti-Flg antibodies (used at a dilution of 1:1000) or mouse monoclonal anti-RBD antibodies (XR06, Xema, Moscow, Russia) (used at a dilution of 1:5000) for 1 h at room temperature. Then, the membrane was washed three times with TBS-T buffer (15 min at room temperature) and incubated with the secondary rabbit anti-mouse antibodies conjugated with peroxidase (P-RAM Iss, Imtek, Moscow, Russia) for 1 h at room temperature. Then, the membrane was washed three times with TBS-T buffer (15 min at room temperature). Specific protein-antibody complexes were visualized using a Western Blot ECL Plus kit (GE Healthcare, New York, NY, USA) and chemiluminescence detector imager Fusion Solo X (Vilber, Eberhardzell, Germany).

### 4.3. Purification of Plant-Produced Proteins Using Metal Affinity Chromatography

The plant-produced RBD-4M2e, Flg-RBD, Flg-RBD-4M2e proteins were purified by metal affinity chromatography using Ni-NTA resin (Qiagen, Hilden, Germany) under denaturing conditions. The *N. benthamiana* leaves were homogenized in buffer containing 6 M guanidine-HCI, 50 mM NaH_2_PO_4_, 300 mM NaCI (pH 8.0) and then centrifuged at 14,000× *g* for 15 min. The supernatant was applied to Ni-NTA resin, the mixture was incubated with agitation for 1 h at room temperature. For RBD-4M2e, the resin was washed twice with the buffer (8 M urea, 50 mM NaH_2_PO_4_, 300 mM NaCI, pH 8.0) containing 10 mM and 20 mM imidazole. For Flg-RBD, Flg-RBD-4M2e the resin was washed six times: three times with the buffer containing 6 M urea, 50 mM NaH_2_PO_4_, 300 mM NaCI, 10 mM imidazole and three times with the buffer containing 6 M urea, 50 mM NaH_2_PO4, 300 mM NaCI, 16 mM imidazole. The recombinant proteins were eluted with buffer containing 4 M urea, 50 mM NaH_2_PO_4_, 300 mM NaCI, and 500 mM imidazole.

The RBD protein was purified on Ni-NTA resin under native conditions. The *N. benthamiana* leaves were homogenized in a solution containing 50 mM NaH_2_PO_4_, 300 mM NaCI (pH 8.0) and then centrifuged at 14,000× *g* for 15 min. The supernatant was applied to Ni-NTA resin, the mixture was incubated with agitation for 1 h at room temperature. The resin was washed twice with the buffer (50 mM NaH_2_PO_4_, 300 mM NaCI, pH 8.0) containing 10 mM and 20 mM imidazole. The recombinant proteins were eluted with buffer containing 50 mM NaH_2_PO_4_, 300 mM NaCI, and 500 mM imidazole.

After elution, the protein samples were dialyzed three times against PBS (1:100) using Slide-A-Lyzer Mini dialysis units (Thermo Fisher Scientific, Waltham, MA, USA). Protein amounts were measured using a Qubit Protein Assay Kit using Qubit Fluorometer and relevant protocols (Invitrogen, Waltham, MA, USA).

### 4.4. Glycoprotein Detection

The presence of glycans in plant-produced purified RBD-4M2e, Flg-RBD, Flg-RBD-4M2e was detected by Pro Q ^®^ Emerald 300 Glycoprotein Gel and Blot Stain Kit (P21857) (Invitrogen, Waltham, MA, USA). About 500 ng of the plant-produced RBD-4M2e, Flg-RBD, Flg-RBD-4M2e was separated on a 10% SDS-PAGE gel, and then the proteins were transferred onto a Hybond-P membrane. The glycans were detected onto the membrane using Pro-Q Emerald 300 glycoprotein staining according to the manufacturer’s protocol.

## 5. Conclusions

This study demonstrated that RBD and RBD-M2e proteins, as well as their fusions with flagellin of *S. typhimurium,* could be efficiently produced in plants using a transient expression system based on the viral vector pEff. The targeting of recombinant proteins to the endoplasmic reticulum enabled the production of the RBD in glycosylated form shown to be critical for the correct folding and functionality of recombinant RBD variants. The plant-produced Flg-RBD-4M2e protein could be further used for the development of recombinant bivalent vaccines against influenza A and COVID-19 that could be delivered intranasally. Moreover, due to the presence of the conserved M2e peptide, such a vaccine is expected to be effective against a wide range of influenza A strains. The induction of the immune response in the nasal compartment is particularly important for vaccines against influenza and COVID-19 since it can locally prevent the infection and the spread of the disease.

## Data Availability

Not applicable.
